# Transient Conformations Leading to Peptide Fragment Ion [c + 2H]^+^ via Intramolecular Hydrogen Bonding Using MALDI In-source Decay Mass Spectrometry of Serine-, Threonine-, and/or Cysteine-Containing Peptides

**DOI:** 10.3390/molecules28237700

**Published:** 2023-11-22

**Authors:** Mitsuo Takayama

**Affiliations:** Graduate School in Nanobioscience, Yokohama City University, 22-2 Seto, Kanazawa-ku, Yokohama 236-0027, Japan; takayama@yokohama-cu.ac.jp

**Keywords:** MALDI-ISD, hydrogen atom transfer, hydrogen bonding, [c + 2H]^+^ ion, Ser/Thr/Cys-based peptides, type II reaction

## Abstract

The formation of a peptide fragment ion [c + 2H]^+^ was examined using ultraviolet matrix-assisted laser desorption/ionization in-source decay mass spectrometry (UV/MALDI-ISD MS). Unusually, an ISD experiment with a hydrogen-abstracting oxidative matrix 4-nitro-1-naphthol (4,1-NNL) resulted in a [c + 2H]^+^ ion when the analyte peptides contained serine (Ser), threonine (Thr), and/or cysteine (Cys) residues, although the ISD with 4,1-NNL merely resulted in [a]^+^ and [d]^+^ ions. The [c + 2H]^+^ ion observed could be rationalized through intramolecular hydrogen atom transfer (HAT), like a Type-II reaction via a seven-membered conformation involving intramolecular hydrogen bonding (HB) between the active hydrogens (–OH and –SH) of the Ser/Thr/Cys residues and the backbone carbonyl oxygen at the adjacent amino (N)-terminal side residue. The ISD of the Cys-containing peptide resulted in the [c + 2H]^+^ ions, which originated from cleavage at the backbone N-Cα bonds far from the Cys residue, suggesting that the peptide molecule formed 16- and 22-membered transient conformations in the gas phase. The time-dependent density functional theory (TDDFT) calculations of the model structures of the Ser and Cys residues indicated that the Cys residue did not show a constructive bond interaction between the donor thiol (-SH) and carbonyl oxygen (=CO), while the Ser residue formed a distinct intramolecular HB.

## 1. Introduction

**Scheme 1:**
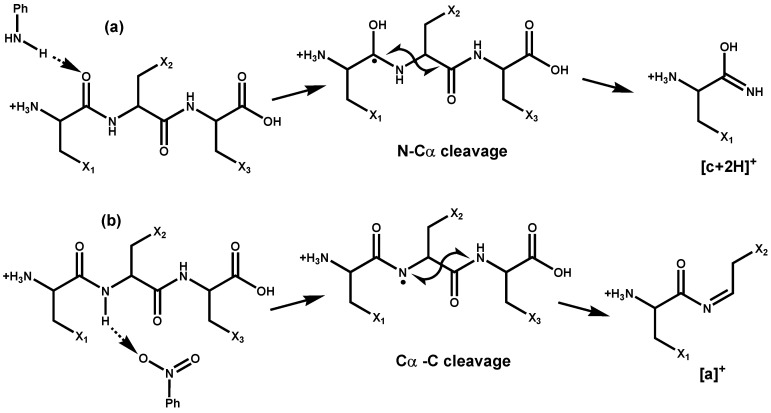
(**a**) Reduced ISD for generating [c + 2H]^+^ using a hydrogen-donating matrix (Ph-NH_2_) [9,10,11,12] and (**b**) oxidized ISD for generating [a]^+^ ions using a hydrogen-abstracting matrix (Ph-NO_2_) [13,14], via intermolecular hydrogen bonding between matrix and peptide molecules. The dashed arrow represents the direction of hydrogen transfer via hydrogen bonding.

**Scheme 2:**
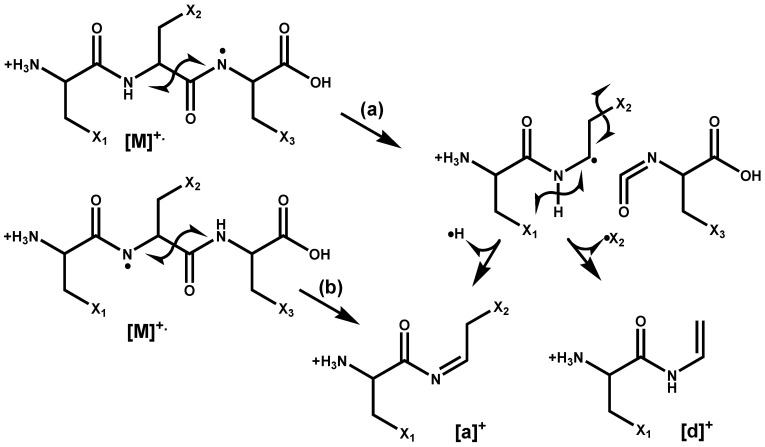
Radical-induced dissociation pathways for the formation of [a]^+^ and [d]^+^ ions in the oxidized ISD [13,14]. (**a**) Pathway for both [a]^+^ and [d]^+^ ions via a radical species originating from cleavage at the Cα-C bond. (**b**) Pathway for only [a]^+^ ion.

Hydrogen atom transfer (HAT) via hydrogen-bonding (HB) plays an essential role in basic and applied chemical interests in a wide variety of photo-induced reactions [1,2,3,4] and in the fragmentation in mass spectrometry (MS) [5,6]. HAT results in radical-induced dissociation (RID) reactions, such as McLafferty rearrangement [5,6] and in-source decay (ISD) coupled with ultraviolet matrix-assisted laser desorption/ionization MS (UV/MALDI MS) [7,8]. The UV/MALDI-ISD is a practical method for the top-down sequencing of peptides and proteins [7,8]. The ISD is a method based on the intermolecular HAT, which occurs by HB between the peptide backbone amide region (–CO-NH–) and the functional groups of the matrix materials for MALDI experiments [9]. Furthermore, the ISD can be classified into two categories, namely reduced ISD and oxidized ISD, according to the chemical properties of the matrix functional groups, such as hydrogen-donating phenolic and anilinic groups (Ph-OH and Ph-NH_2_) [9,10,11,12], and as hydrogen-abstracting nitro (Ph-NO_2_) and carbonyl (Ph-CO–) groups [13,14], respectively. Both reduced and oxidized ISD processes occur within several tens of ns via intermolecular collisional interactions between the matrix and peptide/protein molecules electronically and vibrationally excited with UV photon absorption. It was known that the electronic excitation of matrix molecules caused by UV photon absorption results in the formation of protons, free electrons, abundant hydrogen atoms, peptide fragment ions, and the hydrogen-excess molecular ion [M + 2H]^+^· due to intermolecular HAT [15,16,17,18]. The reduced ISD of peptides and proteins results in a preferential fragment ion [c + 2H]^+^ originating from cleavage at the N-Cα bond between amide nitrogen (N) and alpha carbon (Cα) of the peptide backbone, while the oxidized ISD results in [a]^+^ and [d]^+^ ions, as illustrated in Scheme 1 and Scheme 2. Here, I have obeyed the proposed nomenclature for peptide fragment ions [19]. It is important to recognize that both the reduced and oxidized ISDs occur via pre-formed intermolecular HB between the backbone amide region and the functional group of matrices (see the left-hand side of Scheme 1) [9]. 

**Scheme 3:**
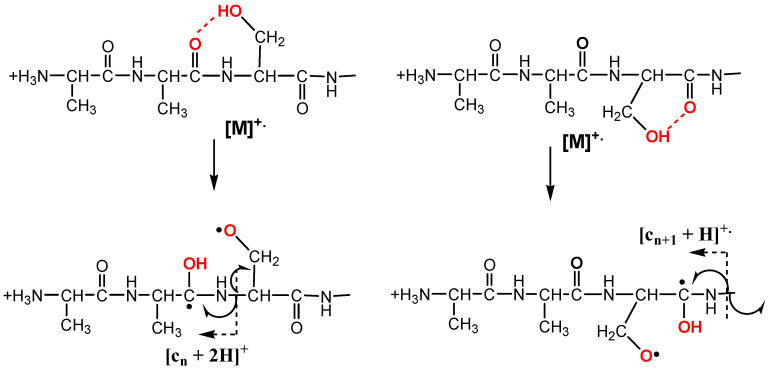
Two possible mechanisms for the formation of [c + 2H]^+^ and [c_n+1_ + H]^+^· ions produced by RID via seven- and six-membered intramolecular hydrogen-bonding at the N-terminal (**left**) and C-terminal side (**right**).

Thomas et al. reported that when using collision-induced dissociation (CID) coupled with an ion-trap mass spectrometer, the CID spectra of peptide radical cations [M]^+.^ resulting in unusual fragment ions, such as [c + 2H]^+^ and [a + H]^+.^ Ions, when analyte peptides contained serine (Ser) and/or threonine (Thr) residues [20]. They demonstrated that the a [c + 2H]^+^ ion could be formed by donating and abstracting hydrogen via intramolecular HB between the hydroxy group (–OH) of Ser/Thr residues and the backbone region. Interestingly, the radical-induced cleavage at the backbone N-Cα bond to form the [c + 2H]^+^ ion was asymmetric with respect to the amino (N)- and carboxyl (C)-terminal sides of the Ser/Thr residues, despite the Ser/Thr hydroxy groups making it possible to form HB, with both side carbonyl groups via seven- and six-membered transition states, respectively, as shown in Scheme 3, although the six-membered hydrogen-bonding conformers (Scheme 3, right) have often been proposed in the condensed-phase study of Ser-based peptides [21]. Although the formation of the biradical species in Scheme 3 could be strongly supported from Type-II reactions [1], the CID spectra did not show the [c_n+1_ + H]^+^· ion originating from the C-terminal N-Cα bond cleavage [20]. Based on an energy diagram based on DFT calculations, Thomas et al. also described that both N-terminal seven- and C-terminal six-membered transition states via the intramolecular HB of Ser/Thr residues favored only the N-terminal N-Cα bond cleavage in order to form a [c + 2H]^+^ ion. We recently reported that some peaks may be assigned as [c + 2H]^+^ ions in the oxidized ISD experiments of peptides even when a hydrogen-abstracting oxidative matrix 4-nitro-1-naphthol (4,1-NNL) was used [22], while the oxidized ISD of the peptides using 4,1-NNL merely yielded [a]^+^ and [d]^+^ ions originating from cleavage at the Cα-C bond [23]. Fukuyama et al. also reported that when using the oxidized ISD with 3-hydroxy-4-nitrobenzoic acid as a matrix, the [c + 2H]^+^ ion peaks could be seen as well as a number of [a]^+^ ion peaks, without mechanistic considerations for the [c + 2H]^+^ ion [24]. Based on the fact that the peptides used for the oxidized ISD experiments, as described above, contained Ser, Thr, and/or cysteine (Cys) residues [22,23,24], it was likely that the oxidized ISD experiments would result in [c + 2H]^+^ ions when the analyte peptides contained Ser, Thr, and/or Cys residues. The hydroxy and thiol active hydrogens (-OH and -SH) of Ser/Thr/Cys residues were capable of donating through HB to acceptors such as the lone-pair electrons of the oxygen, nitrogen, and sulfur atoms. This suggested that the reduced ISD could occur through the formation of intramolecular HB or right transient conformations, which would enable effective intramolecular HAT processes to occur, even under oxidative ISD conditions with an oxidative matrix. 

Here, I report on ISD experiments showing the definite formation of the [c + 2H]^+^ ion originating from cleavage at the backbone N-Cα bond of Ser, Thr, and Cys residues, using systematically synthesized peptides. It was confirmed that the [c + 2H]^+^ ion was formed by the intramolecular HAT from the active hydrogens (–OH and –SH) of Ser/Thr/Cys residues to the backbone carbonyl oxygen of the N-terminal side before the residue, and also the residues far from Ser/Cys residues. It also was proved, especially, that the [c + 2H]^+^ ion observed in the oxidized ISD of the Cys-containing peptide could be produced by the intramolecular HAT via 16- and 22-membered transient conformations involving intramolecular HB. The DFT and time-dependent (TD) DFT calculations were performed to estimate the intramolecular HB of the models of Ser/Thr/Cys residues. 

## 2. Results and Discussion

### 2.1. The [c + 2H]^+^ Ion Formation of a Ser-Based Peptide in Oxidative ISD with a Hydrogen-Abstracting Oxidative Matrix 

**Figure 1:**
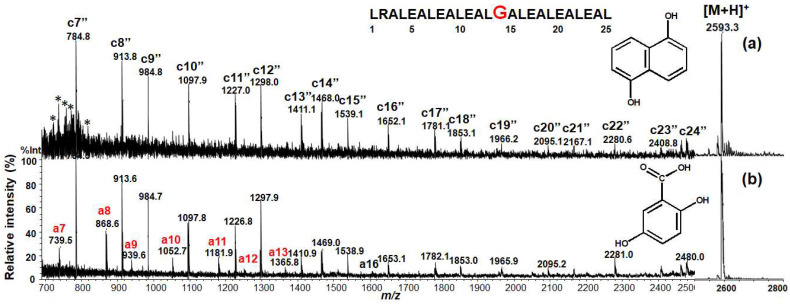
The oxidized ISD spectra of synthetic peptideLRAGly14 obtained with (**a**) hydrogen donating matrix 1,5-DHN and (**b**) hydrogen donating/abstracting matrix 2,5-DHB. Asterisk shows matrix peaks.

**Scheme 4:**
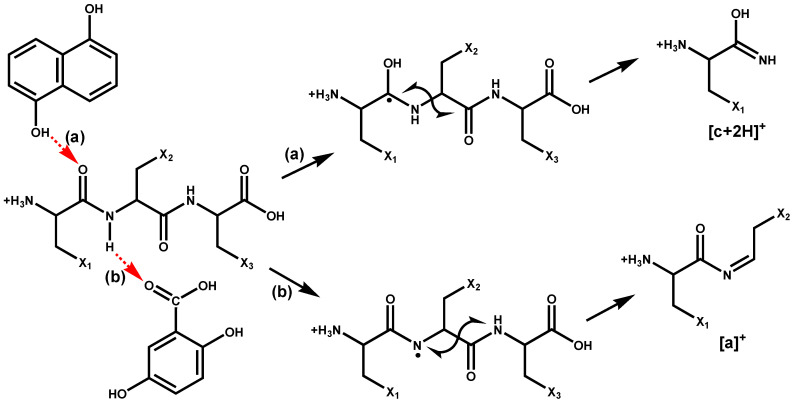
(a) The reduced ISD and (b) oxidized ISD processes for the formation of [c + 2H]^+^ and [a]^+^ ions, using a hydrogen-donating matrix (1,5-DHN) and a dual hydrogen donating/accepting matrix (2,5-DHB). The dashed arrow represents the direction of hydrogen transfer via hydrogen bonding.

First of all, the typical reduced ISD spectra of a Ser/Thr/Cys free peptide LRAGly14 obtained with 2 different hydrogen-donating reductive matrices, 1,5-dihydroxy naphthalene (1,5-DHN) and 2,5-dihydroxy benzoic acid (2,5-DHB), were compared, as shown in Figure 1. Although the spectra preferentially showed the fragment ion peaks corresponding to the [c + 2H]^+^ ion originating from cleavage at the N-Cα bond of the peptide backbone, occurring through HAT from the matrix to the backbone carbonyl oxygen (Scheme 1a), the use of 2,5-DHB resulted in some [a]^+^ ion peaks (Figure 1b). As was already reported [13,14,25], the functional carbonyl group of the matrix compounds played a hydrogen-abstraction role in the backbone amide region of the peptides to form the [a]^+^ ion, as illustrated in Scheme 4b. The 2,5-DHB has a dual capability of hydrogen donating and accepting due to the presence of a hydrogen donor (Ph-OH) and acceptor (Ph-CO-). In the absence of hydrogen-abstracting groups, such as carbonyl and nitro groups in the matrix compounds, the ISD spectra of peptides produced few [a]^+^ ions (i.e., Figure 1a), with 1,5-DHN merely showing the [c + 2H]^+^ ion. Figure 1b shows that the presence of a carbonyl group (even in a carboxyl group) as a hydrogen acceptor was noteworthy at all events involving inter-molecular HB, as shown in Scheme 4. 

**Figure 2:**
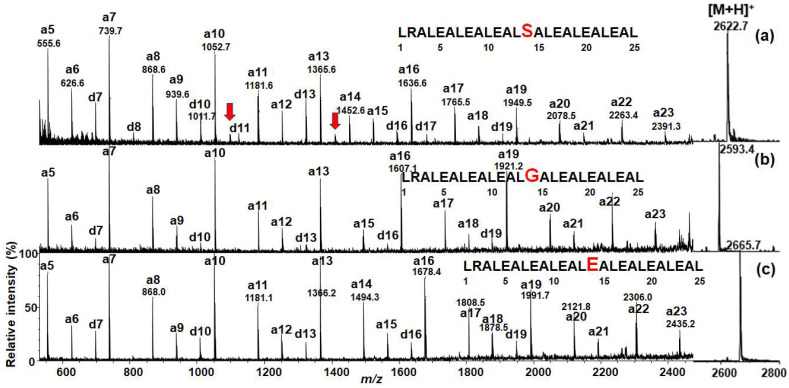
The oxidized ISD spectra of synthetic peptides with (**a**) LRASer14, (**b**) LRAGly14, and (**c**) LRAGlu14, obtained with hydrogen-abstracting oxidative matrix 4,1-NNL. The red arrow in (**a**) shows the peaks assigned as the [c + 2H]^+^ ions.

**Figure 3:**
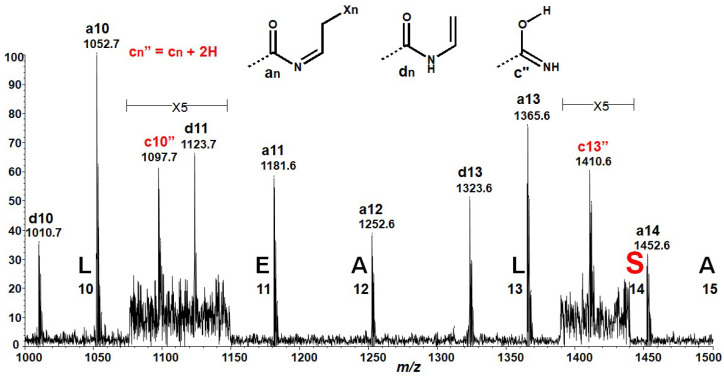
The expanded oxidized ISD spectrum of Ser with peptide LRASer14, obtained with hydrogen-abstracting matrix 4,1-NNL. The inset shows the structures of [a]^+^, [d]^+^, and [c + 2H]^+^ ions.

**Scheme 5:**
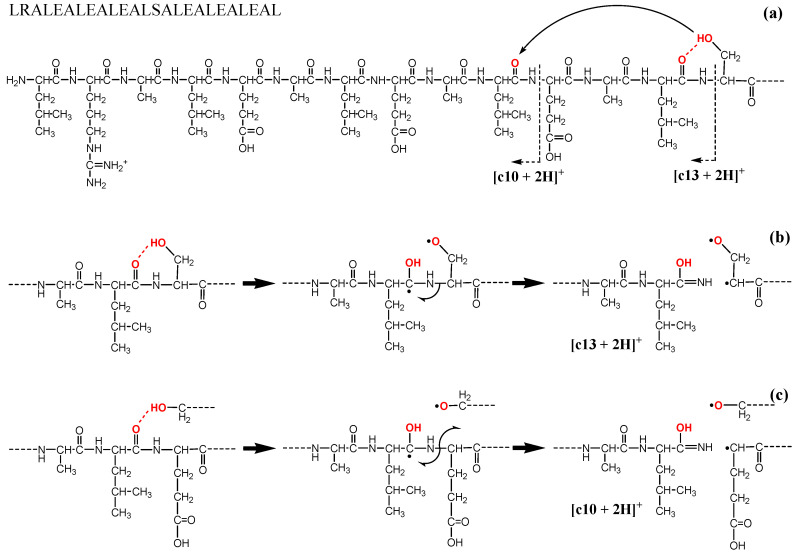
(**a**) The proposed mechanism of the formation of [c13 + 2H]^+^ and [c10 + 2H]^+^ ions by intramolecular HAT from the hydroxyl group of the Ser14 residue to the backbone carbonyl oxygens of the Leu13 and Leu10 residues, the stepwise mechanism for the formation of the (**b**) [c13 + 2H]^+^ and (**c**) [c10 + 2H]^+^ ions.

Figure 2 shows the oxidized ISD spectra of 3 synthetic peptides, LRASer14, LRAGly14, and LRAGlu14, which differ in the 14th amino acid residue (Ser14, Gly14, Glu14), obtained using a hydrogen-abstracting oxidative matrix 4,1-NNL. All the spectra showed [a]^+^ and [d]^+^ ions originating from cleavage at the backbone Cα-C bond and the loss of a side chain from the [a]^+^ ion, respectively, as shown in Scheme 2. Interestingly, the ISD spectrum of the Ser14-containing peptide LRASer14 showed peaks at *m*/*z* 1098 and 1411, corresponding to the [c10 + 2H]^+^ and [c13 + 2H]^+^ ions originating from cleavage at the N-Cα bond of the Leu10-Glu11 and the Leu13-Ser14 residues, respectively, as shown in Figure 3. In Figure 2b, the ISD spectrum of the Gly14-containing peptide was lacking in [a14]^+^ ions, originating from cleavage at the Cα-C bond of the Gly14-Ala15 residues, as already reported [13,14]. Although the [c10 + 2H]^+^ ion at *m*/*z* 1098 originating from the cleavage at the Leu10-Glu11 residues was shown in Figure 3, the ISD spectrum of Glu14-containing peptide LRAGlu14 did not show the [c13 + 2H]^+^ ion originating from the cleavage at the Leu13-Glu14 residues (Figure 2c). This showed that the [c10 + 2H]^+^ ion observed in Figure 3 was not formed by the presence of the Glu11 residue but by the presence of the Ser14 residue. This was of interest from the standpoint of the source of the hydrogen, because the backbone carbonyl oxygen of the Leu10 residue far from the Ser14 residue and the formation of the [c10 + 2H]^+^ ion at *m*/*z* 1098 needed an intramolecular HAT via a 16-membered transition state between the hydroxyl hydrogen of the Ser14 residue and the carbonyl oxygen of the Leu14 residue, as shown in Scheme 5a. Although the formation of the [c13 + 2H]^+^ ion at *m*/*z* 1411 could be easily explained by intramolecular HAT via a seven-membered transition state (Scheme 5b), the formation of the [c10 + 2H]^+^ ion at *m*/*z* 1098 required a suitable conformation of the peptide molecule for the HAT process to occur. Interestingly here, the N-Cα bond cleavage to form [c + 2H]^+^ ions was asymmetric with respect to both the N- and C-terminal sides of the residue of interest, i.e., the N-Cα bond cleavage at the N-terminal side of the Ser residue, as occurred in Scheme 3 (left) and as shown in Figure 3 and Scheme 5. It should be emphasized that the formation of the biradical species in Scheme 3 (left) and Scheme 5b could be rationalized by a UV-induced Type-II reaction, which was a photochemical, intramolecular hydrogen abstraction via a seven-membered transition state of ketones [1]. 

### 2.2. Influence of Cys Residue on the [c + 2H]^+^ Ion Formation

**Figure 4:**
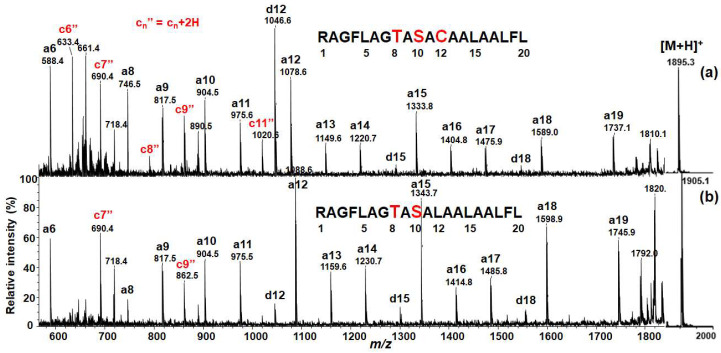
The oxidized ISD spectra of the synthetic peptides (**a**) RAGThr8Ser10Cys12 and (**b**) RAGThr8Ser10, obtained with the 4,1-NNL matrix.

**Scheme 6:**
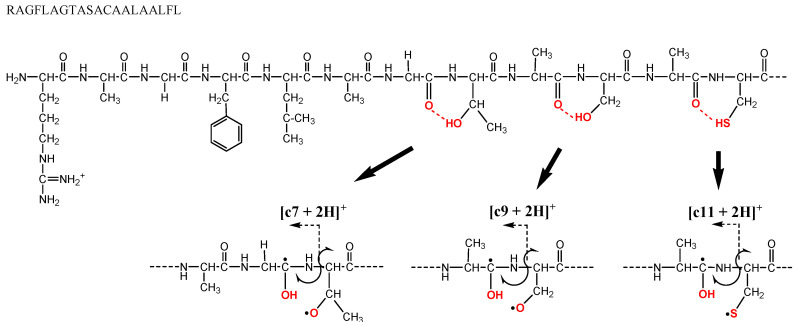
Proposed mechanism for the formation of the [c + 2H]^+^ ions via intramolecular hydrogen bonding between the backbone carbonyl oxygen and the active hydrogens of the side chain of Thr, Ser, and Cys residues of the peptide RAGThr8Ser10Cys12.

To further confirm the influence of the active hydrogen upon the [c + 2H]^+^ ion formation, as described above, 2 different synthetic Ser/Thr/Cys-based peptides, RAGThr8Ser10 and RAGThr8Ser10Cys12, were used to examine the influence of Cys residue in the oxidized ISD with 4,1-NNL. The ISD spectrum of the Cys-containing peptide RAGThr8Ser10Cys12 showed relatively intense peak heights of [c + 2H]^+^ ions, i.e., [c6 + 2H]^+^ at *m*/*z* 633, [c7 + 2H]^+^ at *m*/*z* 690, [c8 + 2H]^+^ at *m*/*z* 792, [c9 + 2H]^+^ at *m*/*z* 862, [c9 + 2H]^+^ at *m*/*z* 949, and [c11 + 2H]^+^ at *m*/*z* 1021, as shown in Figure 4a, while the spectrum of RAGThr8Ser10 simply showed two [c7 + 2H]^+^ and [c9 + 2H]^+^ ions originating from cleavage at the N-Cα bond of Thr and Ser residues (Figure 4b). Although the formation of the [c7 + 2H]^+^, [c9 + 2H]^+^, and [c11 + 2H]^+^ ions of both peptides could be easily explained by the intramolecular HAT via the seven-membered transition state involving HB without an assumption of any complex conformations of peptide molecules, as shown in Scheme 6, it seemed to be difficult to explain the formation of the [c6 + 2H]^+^ ion at *m*/*z* 633 and the [c8 + 2H]^+^ ion at *m*/*z* 792 originating from cleavage at the N-Cα bond of the Ala6-Gly7 and Thr8-Ala9 residues, respectively. 

**Scheme 7:**
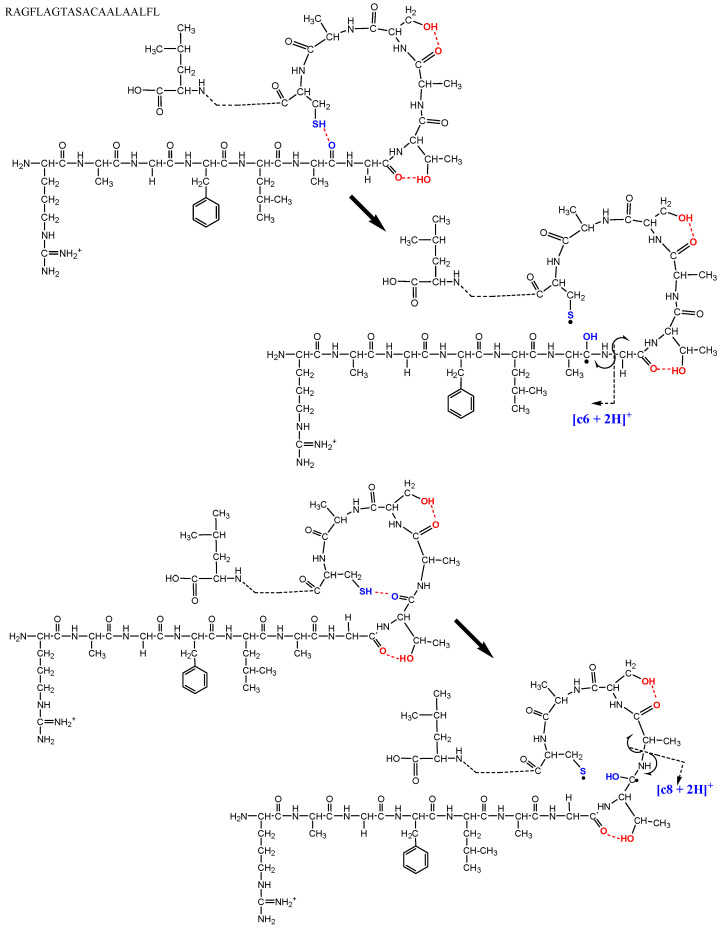
Proposed 22- (**upper**) and 16-membered (**lower**) transient conformations of the peptide RAGThr8Ser10Cys12 for the formation of the [c6 + 2H]^+^ and [c8 + 2H]^+^ ions.

Comparing the oxidized ISD spectra of the peptides with and without Cys residue in Figure 4, it could be presumed that the [c6 + 2H]^+^, [c8 + 2H]^+^, and [c11 + 2H]^+^ ions could be formed by the presence of the Cys12 residue. In particular, it should be noted that the sites of the N-Cα bond of the Ala6-Gly7 and Thr8-Ala9 residues to produce [c6 + 2H]^+^ and [c8 + 2H]^+^ ions were far from the Cys12 residue for forming the intramolecular HB. Although it was difficult to decide on the precise structures of the conformations of the peptides in the gas phase, it was possible to propose that the [c + 2H]^+^ ions could be generated from appropriate conformations that enabled the intramolecular HAT to occur. The flexibility of the peptide molecules might temporarily form the right conformations. Here, I supposed two transient conformations for explaining the [c6 + 2H]^+^ and [c8 + 2H]^+^ ions that were formed by the intramolecular HAT from the thiol (–SH) of the Cys12 residue to the amide carbonyl oxygens (–CO-NH_2_–) of the Ala6-Gly7 and Thr8-Ala9 residues, respectively, as shown in Scheme 7. With respect to the intramolecular HAT used to form [c]^−^ and [y]^−^ ions, we had reported that in a negative-ion CID study of deuterated-peptides, at least three-, four-, six-, seven-, eight-, and nine-membered ring conformations could be transiently formed due to the flexibility of gas-phase peptides [26]. Therefore, it was reasonable to propose that the intramolecular HAT of the peptide RAGThr8Ser10Cys12 could produce the [c6 + 2H]^+^ and [c8 + 2H]^+^ ions through the 22- and 16-membered transient conformations involving intramolecular HB. Consequently, the side chain of the Cys residue could more flexibly interact with the backbone carbonyl oxygen via transient conformations than that of the Ser and Thr residues. The [c + 2H]^+^ ion formation at the sites remote from Ser/Cys residues, as observed in Figure 2a and Figure 4a, indicated that, in general, the gas-phase peptide molecules flexibly changed in the conformation involving transient intramolecular HB and HAT, leading to the formation of radicals and the radical-initiated cleavages, such as the ISD reactions for the formation of the [c + 2H]^+^ ions. With respect to the Cys-containing peptides, it should be noted that unexpected disulfide bridges were rarely formed intra- and inter-molecularly during sample preparation [27,28]. The Cys-containing peptide RAGThr8Ser10Cys12 used here did not form oxidized dimeric products during the sample preparations by means of the mass spectral evidence. 

**Figure 5:**
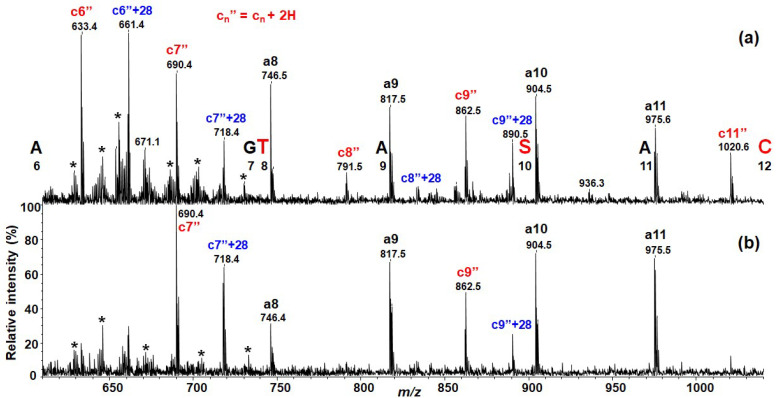
The expanded oxidized ISD spectra of (**a**) RAGThr8Ser10Cys12 and (**b**) RAGThr8Ser10, obtained with the 4,1-NNL matrix. The asterisk represents matrix ion peak.

**Scheme 8:**
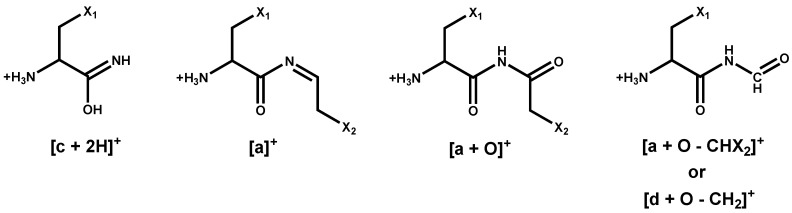
Proposed structure (right-hand side) of the [c + 2H + 28]^+^ ion compared with the structures of [c + 2H]^+^, [a]^+^, and [a + O]^+^ ions.

Next, I will describe the unexpectedly intense peaks corresponding to the [c + 2H + 28]^+^ ions at *m*/*z* 661, 718, 820, and 891, observed in the oxidized-ISD spectra of RAGThr8Ser10 and RAGThr8Ser10Cys12 (Figure 4 and Figure 5). These ion peaks were accompanied by the [c + 2H]^+^ ion, independent of the presence of Cys residue. Since the reductive ISD spectra with a hydrogen-donating matrix did not show such unexpected ions, as shown in Figure 1, it suggested that the [c + 2H]^+^ and [c + 2H + 28]^+^ ions were competitively produced by associating with the intramolecular HAT and some action(s) of the hydrogen-abstracting matrix. Regarding this, I had reported that the oxidative ISD of the peptides resulted in an oxidative product ion [a + O]^+^ caused by an attack of the hydroxyl radical (Ho·) as a strong oxidant generated from the nitro group of the matrix [22]. It should be noted, on the other hand, that the mass of the [c + 2H + 28]^+^ ion agreed with that of [a + O − sidechain]^+^ or [d + O·−·CH_2_]^+^ ions, as was demonstrated in Scheme 8. Although the mechanism(s) of the [c + 2H + 28]^+^ ion formation are unclear at present, it was of interest to try to pin down the case from the standpoint of redox chemistry.

### 2.3. Intramolecular Hydrogen Bonding of the Models of Ser, Thr, and Cys Residues 

**Figure 6:**
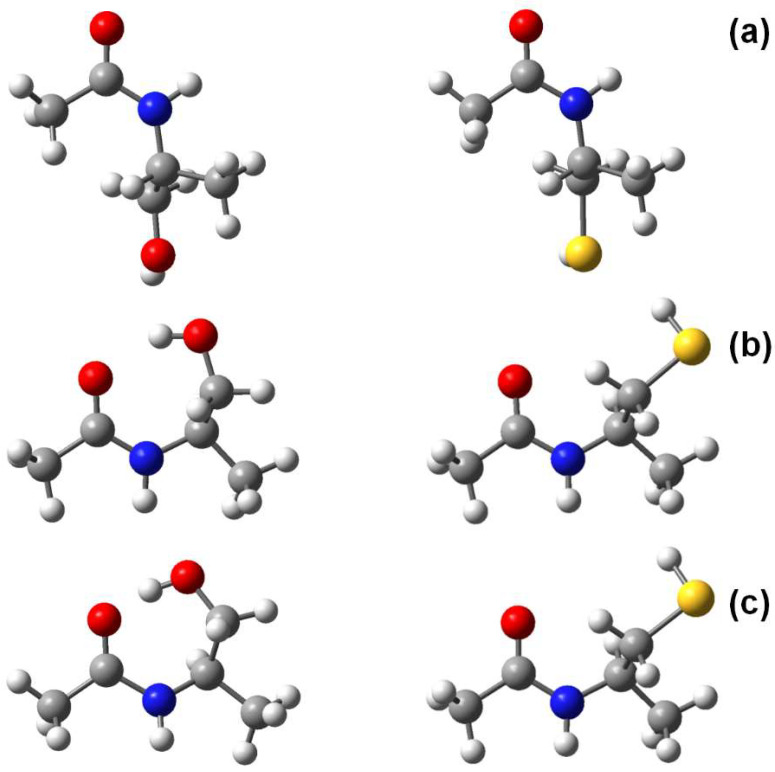
The DFT-optimized structures of the models for Ser (**left**) and Cys (**right**) residues, (**a**) without HB and (**b**) with HB. (**c**) The excited TDDFT-optimized structures of the models for Ser and Cys residues, at the M06-2X/6-311++G(d,p) level. The red, blue, yellow, gray, and white ball-type colors represent oxygen O, nitrogen N, sulfur S, carbon C, and hydrogen H atoms, respectively.

We recently reported that the MALDI-ISD using the 4,1-NNL matrix resulted in over-degraded fragment ions produced from peptide radical ions [M]^+^· [29]. In UV/MALDI experiments, the earliest event was the electronic excitation of the matrix and peptide molecules via UV absorption, which resulted in explosive evaporation to form a dense gas-like MALDI plume [16,17]. Therefore, the [c + 2H]^+^ ion could be produced by photochemical reactions of the excited molecules in the plume. The HAT or proton transfer via the inter/intramolecular HB of excited molecules could be treated using the DFT and TDDFT calculations [30,31,32]. As described in Section 2.2, the Cys residue could more flexibly interact with several carbonyl oxygens at the N-terminal side residues far from the Cys residue, as compared to the Ser and Thr residues. This may have been due to the nature of the weaker HB of the Cys residue, as compared to the Ser and Thr residues. With respect to the capability of the HB donor, it was known that the SH of the Cys residue was nonselective to its hydrogen acceptors, which originated from its weak capability of HB [33]. Here, I performed DFT and TDDFT calculations for the models of the Ser/Thr/Cys residues. The calculations were performed by setting initial model structures with the same conformations of the Ser/Thr/Cys residues, and the structures were fully optimized with respect to the vibrational frequency and energy analysis. The DFT-optimized model structures of the Ser and Cys residues, with and without HB, are shown in Figure 6a,b. Although the optimized structures of the Ser and Cys residues without HB (Figure 6a) were similar in conformation, the Cys residue with HB (Figure 6b) was quite different in conformation from the Ser residue. That is, the hydrogen bond length (HBL) between the thiol hydrogen and carbonyl oxygen atoms of the Cys residue resulted in a long value of 3.96 Å, while HBLs of Ser residue was 1.89 Å. The HBL of the Cys residue was longer than that of the strong (1.2–1.5 Å), moderate (1.5–2.2 Å), and weak (2.2–3.2 Å) hydrogen bonds [34], while that of Ser was in the range of a moderate hydrogen bond. The calculated energies of the models of the Ser/Thr/Cys residues are summarized in Table 1. The stabilization energies ΔE of 18–21 kJ/mol obtained lie in a region of moderate hydrogen bond energies of 17–63 kJ/mol for alcohol and biological molecules [34]. 

It was important to obtain evidence for the presence or the possibility of HB in analyte molecules of interest. The TDDFT calculations were performed on the models of the Ser and Cys residues in order to obtain information on the constructive bond interactions. The TDDFT-optimized structures (Figure 6c) showed that the HB of the Ser residue was in the region of a strong hydrogen bond by changing from 1.89 Å HBL to 1.38 Å upon electronic excitation, while the HBL of the Cys residue did not change. The change in the HBL of the Ser residue was consistent with a Type-II reaction occurring via a photo-induced intramolecular HAT through a seven-membered cyclic structure [1]. The results obtained indicated that the Cys residue did not form sufficient stable or invariable HB, while the Ser residue could form definite intramolecular HB via the seven-membered transition state. Instead, the SH group of the Cys residue may transiently interact with several backbone carbonyl oxygens by forming 7-, 16-, and 22-membered conformations involving intramolecular HB, due to its being nonselective to hydrogen acceptors and its weak capability of HB [33].

## 3. Materials and Methods

### 3.1. Reagents and Sample Preparation

The 4-Nitro-1-naphthol (4,1-NNL), 2,5-dihydroxybenzoic acid (2,5-DHB), and 1,5-dihydroxy naphthalene (1,5-DHN) were purchased from Tokyo Chemical Industry (Tokyo, Japan). Acetonitrile was purchased from Wako Pure Chemicals (Osaka, Japan). Water used in all experiments was purified using a MilliQ water purification system from Millipore (Billerica, MA, USA). All reagents were used without further purification. All synthetic peptides were supplied from the Peptide Institute (Minoh, Osaka, Japan). The primary structures of the synthetic peptides are as follows:

LRASer14 (LRALEALEALEAL**S**ALEALEALEAL, *M*r 2623.0);
LRAGly14 (LRALEALEALEAL**G**ALEALEALEAL, *M*r 2593.0);
LRAGlu14 (LRALEALEALEAL**E**ALEALEALEAL, *M*r 2665.1);
RAGThr8Ser10Cys12 (RAGFLAG**T**A**S**A**C**AALAALFL, *M*r 1895.2); and
RAGThr8Ser10 (RAGFLAG**T**A**S**ALAALAALFL, *M*r 1905.3).

Each peptide was dissolved in water at a concentration of 100 pmol/μL. The matrix was dissolved in water/acetonitrile (1:1, *v*/*v*) without any additives, and the matrix solution was prepared at 10 μg/μL. A sample solution was prepared by mixing a volume of 10 μL of the dissolved analyte peptide with a volume of 10 μL of the matrix solution. A volume of 1.0 μL of the sample solution was deposited onto a stainless plate for the MALDI ion source and the solvents were removed by evaporation in air at room temperature. 

### 3.2. Mass Spectrometry

The ISD spectra with UV/MALDI MS were acquired on a time-of-flight (TOF) mass spectrometer AXIMA-CFR (Shimadzu, Kyoto, Japan) equipped with a nitrogen laser (337 nm wavelength, 4 ns pulse width) operating at a pulse rate of 10 Hz. The laser spot size on the target substrate was ca. 200 μm in diameter. The laser fluence used for 4,1-NNL/peptide systems was 500 J/m^2^. The ions generated by UV/MALDI were accelerated using 20 kV with delayed extraction for a 30 ns delay time. The TOF analyzer was operated in a high-resolution reflectron mode, and the ions were detected using a detector on a microchannel plate. A total of 500 laser shots were accumulated for each mass spectrum acquisition. 

### 3.3. Calculations

The initial model structures of Ser, Thr, and Cys residues, with and without intramolecular HB, were generated by means of visual inspection using the GaussView program 6.0 [35]. The model structures were fully optimized by using the Gaussian 16 suite of programs [35]. The geometry optimization and vibrational frequency analysis were performed using the M06-2X hybrid functional [36] level of theory and the 6-311++G(d,p) basis set. The energies were evaluated from the sum of electronic and zero-point energies calculated by the same level of the theory and basis set. The TDDFT calculations were performed on the optimized structures of Ser and Cys residues. 

## 4. Conclusions

Unusually, oxidized ISD experiments combined with the UV/MALDI MS of systematically synthesized peptides resulted in a peptide fragment [c + 2H]^+^ ion originating from cleavage at the backbone N-Cα bond when the peptides contained Ser, Thr, and/or Cys residues, although the oxidized ISD with the hydrogen-abstracting oxidative matrix 4,1-NNL merely produced peptide fragment [a]^+^ and [d]^+^ ions originating from cleavage at the backbone Cα-C bond. The formation of the [c + 2H]^+^ ion could be explained by the intramolecular HAT via pre-existing intramolecular HB between the OH/SH hydrogens of the Ser/Thr/Cys residues and the backbone carbonyl oxygens at the adjacent N-terminal side residue or the residues far from the Ser/Thr/Cys residues. The [c + 2H]^+^ ions originating from the cleavage at the N-Cα bonds, especially those far from the Cys residue, could be explained by assuming the 16- and 22-membered transient conformations involved intramolecular HB. The quantum chemical DFT and TDDFT approaches indicated that the Ser residue formed a definite 7-membered conformation involving HB to produce the [c + 2H]^+^ ion, which could be rationalized by the Type-II reaction induced by the UV photons, while the Cys residue could have transiently formed the 7-, 16-, and 22-membered conformations involving HB due to its weak HB capability. 

## Figures and Tables

**Table 1:** Stabilization energy ΔE of the model compounds for Ser, Thr, and Cys residues, without and with hydrogen bonding (wo-HB and w-HB), at the M06-2X/6-311++G(d,p) level.

	wo-HB	w-HB	ΔE (kJ/mol)
Ser	−402.145672	−402.153207	−19.78
Thr	−441.426390	−441.434413	−21.06
Cys	−725.117970	−725.124771	−17.86

## Data Availability

All the data are available within the manuscript.
